# The influence of modern living conditions on the human microbiome and potential therapeutic opportunities for allergy prevention

**DOI:** 10.1016/j.waojou.2023.100857

**Published:** 2024-01-03

**Authors:** Guicheng Zhang, Peter Le Souëf

**Affiliations:** a School of Population Health, Curtin University, Perth, 6102, Western Australia, Australia; b School of Biomedical Sciences, The University of Western Australia, Perth, Western Australia 6008, Australia; c School of Medicine, The University of Western Australia, Perth, Western Australia 6008, Australia

**Keywords:** Asthma, Allergy, Microbiome, Modern living conditions, Immigrants

## Abstract

Modern living conditions and the recent surge in global urbanization have transformed the human microbiome. This transformation is believed to be a significant factor in the recent spike of common chronic inflammatory diseases like asthma and allergies worldwide, evident in both developed and developing nations. Immigrants from less developed regions who settle in highly urbanized and affluent areas present an ideal demographic for research. Investigating immigrant populations can yield valuable insights, particularly when studying microbiome changes that occur as individuals transition from areas with low asthma prevalence to regions with a high prevalence of the condition. The application of prebiotics and probiotics as potential treatments for asthma and allergies faces challenges. This is due to the complex interplay of numerous factors that contribute to their aetiology. Exploring the interaction between the human microbiome and potential epigenetic changes in specific populations, such as immigrants adapting to new, urbanized environments, may offer crucial insights. Such research could underscore the role of prebiotics and probiotics in preventing allergic conditions. Recognizing the changes in the human microbiome in the context of a Western/modern environment might be essential in addressing the increasing prevalence of allergic diseases. Persistent research in this domain is pivotal for devising effective interventions such as dietary supplementation with prebiotics and probiotics.

## Introduction

Humans were not necessarily designed to thrive in affluent or western societies.^1^ The intricate exposure to diverse microorganisms, to which our ancestors adapted over the past 60 million years since mammals emerged, has significantly diminished in the past couple of centuries.^2^ This shift has resulted in a misalignment between the environment for which we were originally adapted and our current/modern living conditions. As a result, there has been a recent surge in the prevalence of "Western diseases" or "Modern diseases" worldwide, especially in developed countries.^2^ The impact of modern living conditions on the human microbiome has received considerable attention in research and public discourse as a potential and causal contributor to these diseases. A hygiene hypothesis,^3^ backed by robust research on the benefits of close contact with farm animals,^4^^,^^5^ posits that this disparity has led to atypical reactions to harmless substances/allergens in our immediate surroundings. Human immune system, in the absence of the rich, complex, and regularly challenging microbial load it was developed for, misinterprets harmless substances (allergens), prompting a range of self-destructive diseases like autoimmune disorders and allergic diseases such as asthma and atopic eczema.^6^ This phenomenon is considered a major contributing factor to the high prevalence of these western diseases in developed countries as well as an increasing trend recently in developing countries.

Diverse strategies have been put forth to address the environmental mismatch, with emphasis on both the environment and the human microbiome modifications. There is significant ongoing research on therapeutic interventions focusing on the microbiome and its relation to allergic diseases. Probiotics and prebiotics have been highlighted as a promising avenue in allergy management.^7^ Faecal microbiota transplantation is also being considered as a treatment, but so far, it has not shown notable success in treating allergic disorders.^8^ Despite these efforts, there is still a shortage of proven and reliable microbiome-based interventions that have been conclusively validated and incorporated into clinical practice to prevent allergic diseases. In this brief review, we will discuss various aspects drawing from the latest research on modern living conditions, the human microbiome, and their relation to allergic diseases.

## Urbanization and industrialization

Over centuries, urbanization and industrialization are 2 interconnected processes that have played a significant role in modelling the modern world where we are living in. Evidently, these processes have dramatically reshaped the human environment, and in turn, influenced our exposure to a variety of microbial communities.^9^ Several important factors inherited or accompanied by urbanization and industrialization have apparently been implicated in influencing the microbiomes of both the environment and humans. To list a few of these factors, urbanized environments typically have lower biodiversity than natural environments.^10^ With butterflies as an example, a world-wide recognised allergist with his hobby as a butterfly lover asserted that “Allergy is rare where butterflies flourish in a biodiverse environment”.^11^ Urbanization has disrupted ecosystems and destroyed habitats, leading to the decline and extinction of numerous living organisms.^12^ Observable animals and insects, visible to the naked eye, have been observed vanishing as a result of human activities linked to urbanization and industrialization.^13^ This phenomenon attracts significant attention due to its apparent impact. However, the loss of microorganism species, which are minuscule yet plentiful, often goes unnoticed. From a human health standpoint, it is crucial to closely monitor the changes in microbiome profiles and the reduction in biodiversity occurring in our environment because the entire microbiome represents the “true” environment within which we live and coexist. Industrialized food production and urban dietary habits also affect the human gut microbiome. Diets high in processed foods and low in fibre can lead to a decrease in gut microbial diversity.^14^ The wide use of antibiotics has also drastically altered the human microbiome.^15^ Improvements of quality of drinking water are another important contributing factor to impact on the microbiome exposure of humans over the past one or two centuries.^16^ In addition, the changes of the building environment — homes, workplaces, public transport, etc have re-established own distinct microbiomes in an environment we are in everyday, and the microbiome is often less diverse than those found in natural environments. Reduced exposure to livestock and soil have also potentially limited the diversity of our microbiome exposure.

Apparently, these processes of urbanization and industrialization have resulted in a reduction in microbiome diversity,^17^^,^^18^ which may have significantly contributed to the increase in non-communicable diseases in developed countries in past centuries as well as recently in developing countries. The decreased exposure to a wide range of microorganisms in humans, through its influence on human immune response, explains the disparity in allergic diseases between urban and rural areas and between developed and developing countries. The loss of biodiversity, including microorganism species, due to urbanization and industrialization, is often disregarded, yet it has significant implications for human health. Understanding and documenting the microbiome profiles in both humans and the environment are crucial to safeguard mutual beneficial microorganisms and mitigate the risk of potential global epidemics of common chronic inflammatory diseases.

## Immigrations from low to high resourced countries

Immigrants from low-industrialized countries to highly industrialized countries like Australia represent an ideal population for studying the microbiome changes associated with urbanization and industrialization and possible health relevance. Examining these changes can help uncover potential links between microbiome alterations and the development of “Modern/Western diseases”. Taking allergic diseases such as asthma as an example, the Western environment (or modern urbanization) unquestionably plays a significant role in the development of these conditions. The observations are: 1) during the second half of the 20th century there has been a steep increase in allergic disease in Western developed countries, leading to large allergy contrasts such as between West and East Germany,^19^ Sweden and Estonia,^20^ and Finnish and Russian Karelia;^21^ 2) these differences are apparent between urban and rural regions and suggest that urbanization and the Western lifestyle increase the risks; and 3) migrants that move from developing to developed countries experience a gradual increase in asthmatic and allergic symptoms.^22^ The worldwide pattern of this allergy epidemic was seminally described by the International Study of Asthma and Allergies in Childhood (ISAAC),^23^^,^^24^ the most important epidemiological studies in recent history for asthma and allergy. Based on these seminal studies it can unambiguously be concluded that allergic conditions are higher in developed/high resourced countries and lower in developing/low resourced countries.

Like newborns with ongoing exposure to their environment from birth cohort studies, new immigrants to the western environment provide the unique opportunity to investigate the aetiology of asthma and allergy as well as other western diseases/conditions. Study on new immigrants may hold the key to explain the role of western environmental risk factors for asthma and allergy.

We are focusing on the unique immigrant population from low asthma-risk countries moving to a western environment (Australia). Immigrants from low-risk asthma countries show significant shifts in their immune responses and distinct microbiome profiles, reinforcing the role of environmental microbial exposure in the development of asthma and allergies. In Australian Chinese immigrants, we observed a significant shift in the innate and adaptive immune response, coinciding with adaptation to the western environment.^25^^,^^26^ Toll-like receptor signalling, a crucial innate immune protection pathway against microorganisms and environmental invaders, exhibited a complex U-shaped response over time in these immigrants living in Australia.^27^ We also identified distinct microbiome profiles in Chinese immigrants versus those remaining in their home countries with specific taxa dominant in children with allergies.^28^ Our observations suggest that the increase in allergy in immigrants may result from specific taxa.^28^ We also identified differences in oropharyngeal microbiota in recent compared with long-term adult immigrants from low asthma-risk countries.^29^ In our recent publication, we also found that nasal Staphylococcus pneumoniae may play a role in the development of allergic conditions in Chinese immigrants in a Western environment.^30^ *Staphylococcus aureus* and pneumoniae are species of bacteria that commonly exist in the human upper airway. We found they had higher prevalence in long-term compared with short-term Chinese immigrants with Staphylococcus pneumoniae carriage have a five-fold increased risk of doctor-diagnosed eczema compared to immigrants without Staphylococcus pneumoniae carriage.^30^ In addition, environmental exposure is important for the establishment of human microbiomes. We found Australia and China have a different house dust microbiome profile and modern urbanisation has reshaped the bacterial microbiome profiles of house dust in domestic environments.^9^

## The cross talk between the epigenome and microbiome

We conducted investigations into the significant interactive effects of the Western environment on genetics, specifically regarding atopy and asthma, in various populations. We compared Western/developed populations, such as Finnish Karelians and Inuits based in Copenhagen, with Eastern/developing populations like Russian Karelians and Inuits in Greenland.^31^, ^32^, ^33^, ^34^, ^35^ The findings undoubtedly revealed variations in the genetic effects on asthma and atopy among populations living in contrasting Western and Eastern environments.

Considering that both the epigenome and the microbiome play crucial roles in human health and disease development it is plausible to surmise the interaction between the western environments and genes has underlying epigenome-microbiome mechanisms. Interestingly, we have shown that Chinese immigrants adapting to the Western environment in Australia exhibit time-related changes in genome-wide methylation and gene expression.^36^

**Fig. 1:**
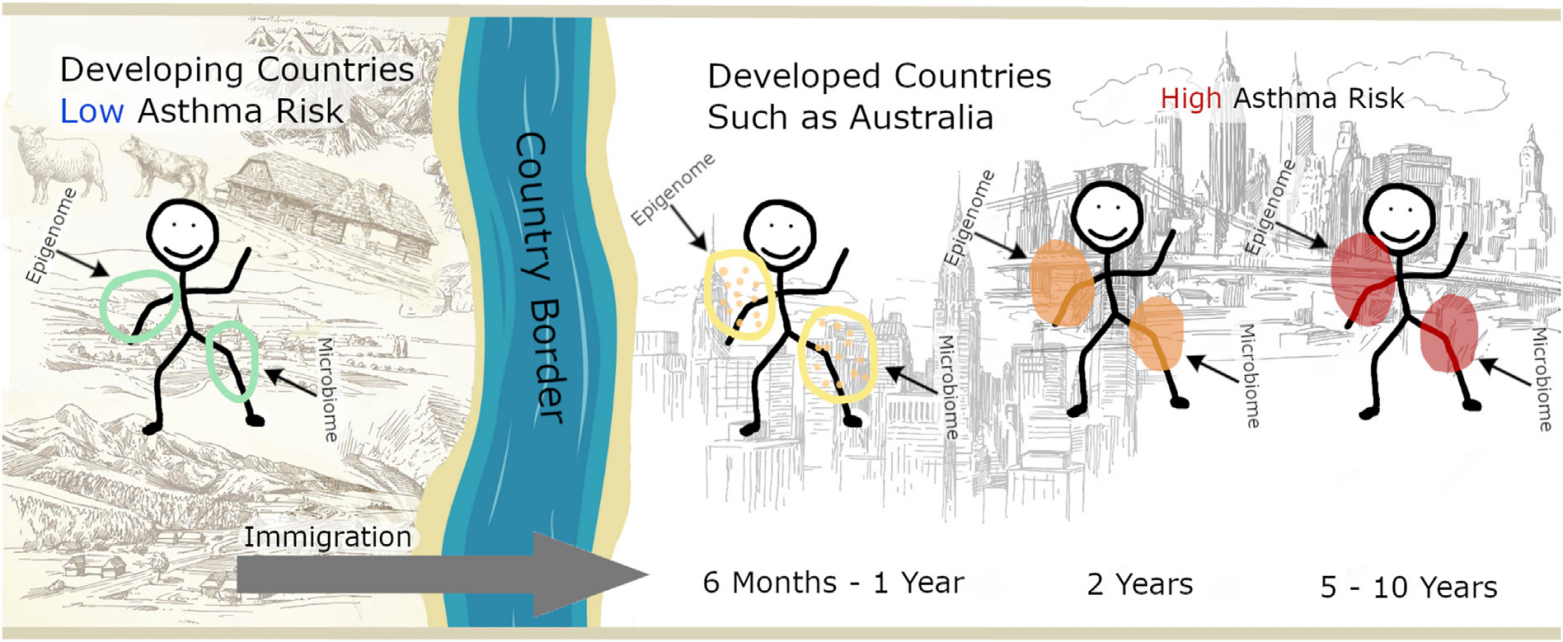
Asthma Risk Evolution in Immigrants: The Potential Role of Epigenetic and Microbiome Alterations Over Time in Developed Countries. On the left side of the figure, a rural or less-developed landscape is shown. On the right, an urban landscape is depicted, representing a highly urbanized environment. The figure demonstrates the increase in asthma risk among populations migrating from developing to developed countries. In the rural setting, the risk of asthma is lower. However, as individuals immigrate to highly urbanized areas in developed countries, the symbols representing the epigenome and microbiome change colour from green to orange, and eventually to red, indicating a heightened risk of asthma. This colour transition signifies potential alterations in the epigenome and microbiome due to new environmental exposures encountered after immigration.

Fig. 1 succinctly illustrates the conceivable epigenome and microbiome changes over time with immigration from an asthma low risk/under resourced to an asthma high-risk countries/developed countries. As the changes in epigenome and microbiome will occur at the same time with immigrants living in a new environment it is expected that the cross talk between the human microbiome and epigenome will have an impact on immune response adaptation and play a crucial role in the development of the western/modern diseases in these immigrants. The mechanism underlying the development of western diseases in immigrants from low-asthma countries may also explain the increase of western diseases in developed countries over the past century.

Emerging evidence suggests that the microbiome can influence the host epigenome,^37^ and conversely, epigenetic modifications can shape the composition and function of the microbiome.^38^ This bidirectional communication between these two systems can have profound implications for human health and the development of various diseases. For example, gut bacteria can interact with immune cells in the gut-associated lymphoid tissue and modulate the production of cytokines and other immune molecules.^39^ The host epigenome can shape the composition and function of the gut microbiome. Epigenetic modifications can indirectly impact the microbial communities residing in the gut.^38^ On the other side, short-chain fatty acids (SCFAs) produced by certain gut bacteria have been shown to influence histone modifications^40^ and DNA methylation patterns,^41^ thereby modulating gene expression in the host. Microbiota-derived metabolites are believed to play a crucial role in regulating the host epigenome. Various microbial signals, including metabolites like butyrate and indoles, bile acids, inflammation, and changes in microbial composition, are thought to contribute to this regulatory process.^42^ Certain non-coding RNAs have been reported to play a part in the responses of intestinal epithelial cells to microbes.^43^ Through affecting the methyl group donor S-Adenosylmethionine (SAM), microbiota also can impact the DNA methyltransferase 2 (DNMT2),^42^ which are writers of the epigenome. There is an urgent need to advance our understanding of the interplay between the epigenome and microbiome, including the identification of specific messenger molecules and determining how gut bacteria influence the epigenome in distant sites not in direct contact with the microbial flora. The gut microbiome's influence on epigenetic modifications is a potential mechanism that may impact human health and disease and studying this relationship in new immigrants will shed light on the development of common western diseases, such as asthma.

## Challenges in microbiome research and therapeutic potential

Research on the microbiome and its role in allergic diseases faces challenges, including inconsistent and contradictory results, false discoveries, and the complexity of the microbiome's association with human health. Despite these challenges, epidemiologic studies have shown that an association exists between environmental exposures that alter the microbiota and the development of atopic dermatitis, food allergies, and asthma.^44^ Clinical trials, considered the gold standard in academic research, are essential for identifying protective microorganisms that can be harnessed to improve human health. Many clinical trials and epidemiological studies have indicated that prebiotics and probiotics have had limited success in allergy management. Further research is needed to determine their effectiveness.^45^ Given the impact of contemporary living conditions on both the environment and the human microbiome, studies comparing different populations and geographical regions could be essential in comprehending the intricate interplay among environmental microbial exposure, genetic predisposition, and epigenetic factors in allergy development.

Until now, clinical trials have not provided conclusive evidence to support the use of probiotics for asthma, food allergies, and other allergic diseases, except for their potential in preventing eczema in high-risk families.^46^ However, for eczema, a recent review assessed the evidence regarding the use of probiotic supplementation for atopic dermatitis prevention in children. It concluded that further studies are needed before the World Allergy Organization (WAO) recommends routine use of such probiotics.^47^ Based on the limited results of clinical trials, WAO recommends the use of probiotics during pregnancy and early infancy to prevent allergy in infants at high allergy risk.^46^ Many clinical trials suffer from inadequate statistical power or a short follow-up duration. To address this issue, consortium efforts are required to achieve more reliable outcomes. Given the complex interplay of various factors in the development of allergic diseases, it is understandable to occasionally feel pessimistic, overwhelmed, and frustrated. In contrast to the hygiene hypothesis, the recent biodiversity hypothesis^48^ suggests that the entire bio-ecosystem might have interactive roles similar to a concert in relation to allergic diseases.^13^ An abnormality or absence of any small component within the ecosystem, particularly in the context of a Western environment, could potentially explain the susceptibility to such diseases.

Overall, while urbanization can reduce certain health risks, however it also poses significant new ones, and the loss of biodiversity with the modern/western environment may have long-term health impacts that we have yet to fully comprehend. The ultimate impact on health depends on a complex mix of factors, including how well urban development is managed, the specific local context, socioeconomic factors, and more.

Over millions of years of co-evolution, commensal bacteria have acquired numerous physiological functions essential to our health, including but not limited to the production of vitamins and the digestion of insoluble dietary fibres. Recent insights into disease origins support a broader range of factors that may predispose, initiate, or exacerbate altered immunity in allergic diseases. These factors include inherent epithelial barrier dysfunction,^49^^,^^50^ loss of immune tolerance at central and specific sites,^51^ and disturbances in gut and organ-specific microbiomes, diet, and age. However, these studies are still in their early stages and have only been considered in a reductionist, disease or tissue-specific manner to date. Only a few studies have explored the cross-talk between various organs, leading to the concept of allergy as a systems disease.^52^ A better understanding of the distinct or shared complex web of factors underlying the spectrum of allergic disorders and the successes/failures of current therapies may pave the way for the development of safer, disease-modifying interventions in the future.

## Conclusion

The impact of modern living conditions on the human microbiome and epigenome, coupled with their interplay in relation to allergic diseases, continues to be a focal point of research. Grasping the complex relationship between the microbiome and the Western environment in terms of human health is essential. Implementing findings on the use of prebiotics and probiotics for asthma and allergy prevention presents challenges. A greater emphasis on extensive and synchronized clinical trials is needed to pinpoint protective prebiotics and probiotics that can aid in allergy prevention. Alterations in the microbiome attributed to contemporary living conditions might offer insights, potentially playing a central role in addressing the rising occurrence of allergic diseases. Continued research in this area is vital to shape effective interventions.

## Abbreviations

WAO, World Allergy Organization; SCFAs, short-chain fatty acids; DNA, Deoxyribonucleic acid; SAM, S-Adenosylmethionine; DNMT2, DNA methyltransferase 2.

## Acknowledgments

We acknowledge the constructive comments from the respiratory research team at Telethon Kids Institute.

## Funds

The study was supported by NHMRC with the grant ID: 2021763.

## Availability of data and materials

N/A.

## Authors contributions

GZ wrote the manuscript and PL contributed to the writing.

## Authors’ consent for publication

GZ and PL are submitting the review manuscript for publication in WAO Journal.

## Ethics approval

RGS0000002369 (Government of Western Australia Department of Health).

## Declaration of competing interest

We declare that we have no conflicts of interest.
